# Unexplained Gastric Ulcer: Role of Cinacalcet

**DOI:** 10.7759/cureus.80226

**Published:** 2025-03-07

**Authors:** Christine Son, Joshua Kalapala, Jeff Leya, Nirmal Desai, Promila Banerjee

**Affiliations:** 1 Internal Medicine, Loyola University Medical Center, Maywood, USA; 2 Gastroenterology, Loyola University Chicago Stritch School of Medicine, Maywood, USA; 3 Gastroenterology, Loyola University Medical Center, Maywood, USA; 4 Gastroenterology and Hepatology, Loyola University Edward Hines Veterans Affairs (VA) Hospital, Hines, USA

**Keywords:** cinacalcet, gastric ulcer, hypercalcemia, peptic ulcer disease (pud), primary hyperparathyroidism (phpt)

## Abstract

Peptic ulcer disease (PUD) most commonly arises as a complication of *Helicobacter pylori* (*H. pylori) *or impaired mucosal defense mechanisms against acid exposure by medications such as non-steroidal anti-inflammatory drugs (NSAIDs). Hypercalcemia can present with peptic ulcer-related symptoms, and cinacalcet is an effective medical treatment for primary hyperparathyroidism (PHPT) for non-surgical candidates. We present a case of a 66-year-old male patient with a history of primary hypercalcemia as a result of hyperparathyroidism, on chronic cinacalcet, who was found to have gastric ulcers on endoscopy. After the reduction of the cinacalcet dose in one year, there was a complete resolution of inflammation in the gastric body.

## Introduction

Peptic ulcer disease (PUD) often occurs in areas exposed to acid and pepsin as a break in the mucosal lining of the stomach or proximal intestine. It most commonly occurs as a complication of *Helicobacter pylori* (*H. pylori*) infection [1]. Medications are the next-most common cause of PUD, specifically nonsteroidal anti-inflammatory drug (NSAID)-associated gastropathy [2]. It is thought that NSAIDs impair prostaglandin-mediated mucosal protection against acidic damage, leading to gastric ulcers [3]. In disorders like Zollinger-Ellison syndrome and antral G-cell hyperfunction, gastrin stimulates gastric acid hypersecretion, leading to peptic ulcer formations. However, the significance of hormonal abnormalities in peptic ulcers with normal gastrin levels is not well understood [4]. Other causes of gastric ulcers are not well defined, and idiopathic gastric ulcers are diagnosed when there is no identifiable cause and ulcers arise spontaneously.

Primary hyperparathyroidism (PHPT) leads to an elevation in parathyroid hormone (PTH) and resulting hypercalcemia. Prior studies have shown that extracellular calcium stimulates gastrin and gastric acid secretion in humans, specifically with oral calcium carbonate ingestion in duodenal ulcer patients [5-9]. The extracellular calcium-sensing receptor (CaSR) on the parathyroid cell surface negatively regulates the PTH secretion. Calcium-sensing receptor is expressed not only in cells secreting calcium-regulating hormones, which are parathyroid cells and thyroid C-cells, but also in cells involved in calcium transport, such as intestinal cells, osteoblasts, and cells of nephron segments [10]. The CaSR expressed on the surface of G cells and parietal cells offers a potential mechanism for calcium’s effect on gastrin and gastric acid secretion and the resulting peptic ulcer formation [11].

Cinacalcet is a drug that acts as a calcimimetic by activating the allosteric site of CaSR [12], thereby mobilizing intracellular calcium stores and inhibiting PTH secretion. Cinacalcet reduces both serum calcium and PTH levels in PHPT [12, 13], serving as an effective medical treatment for non-surgical candidates [13].

This article was previously presented as a meeting abstract at the 2024 American College of Gastroenterology (ACG) Annual Meeting on October 28, 2024.

## Case presentation

A 66-year-old male patient with a history of thyroid cancer status post thyroidectomy and PHPT status post parathyroidectomy, who was treated with cinacalcet for recurrent hypercalcemia, presented with a six-month history of epigastric pain and reflux symptoms. A few months after the symptom onset, he was incidentally found to have increased wall thickening and dilatation of the upper esophagus on an annual low-dose CT chest performed for pulmonary nodule surveillance. An esophagogastroduodenoscopy (EGD) on this occasion revealed mucosa consistent with Barrett’s esophagus, a distal esophageal ulcer, and diffuse erythematous changes in the gastric mucosa. A follow-up EGD was scheduled for eight weeks after treatment of the esophageal ulcer with omeprazole 40 mg twice a day (BID).

The patient initially had chronic parathyroiditis, or chronic inflammation or overactivation of the right superior and inferior parathyroid glands, in 2009. He subsequently underwent removal of the right inferior parathyroid gland with right superior parathyroid autotransplantation. Post-operatively, calcium level was normal; however, PTH continued to be elevated, and hypercalcemia recurred a few months later. The patient was eventually started on cinacalcet 60 mg twice daily in April 2015 for this recurrent hypercalcemia.

**Figure 1:**
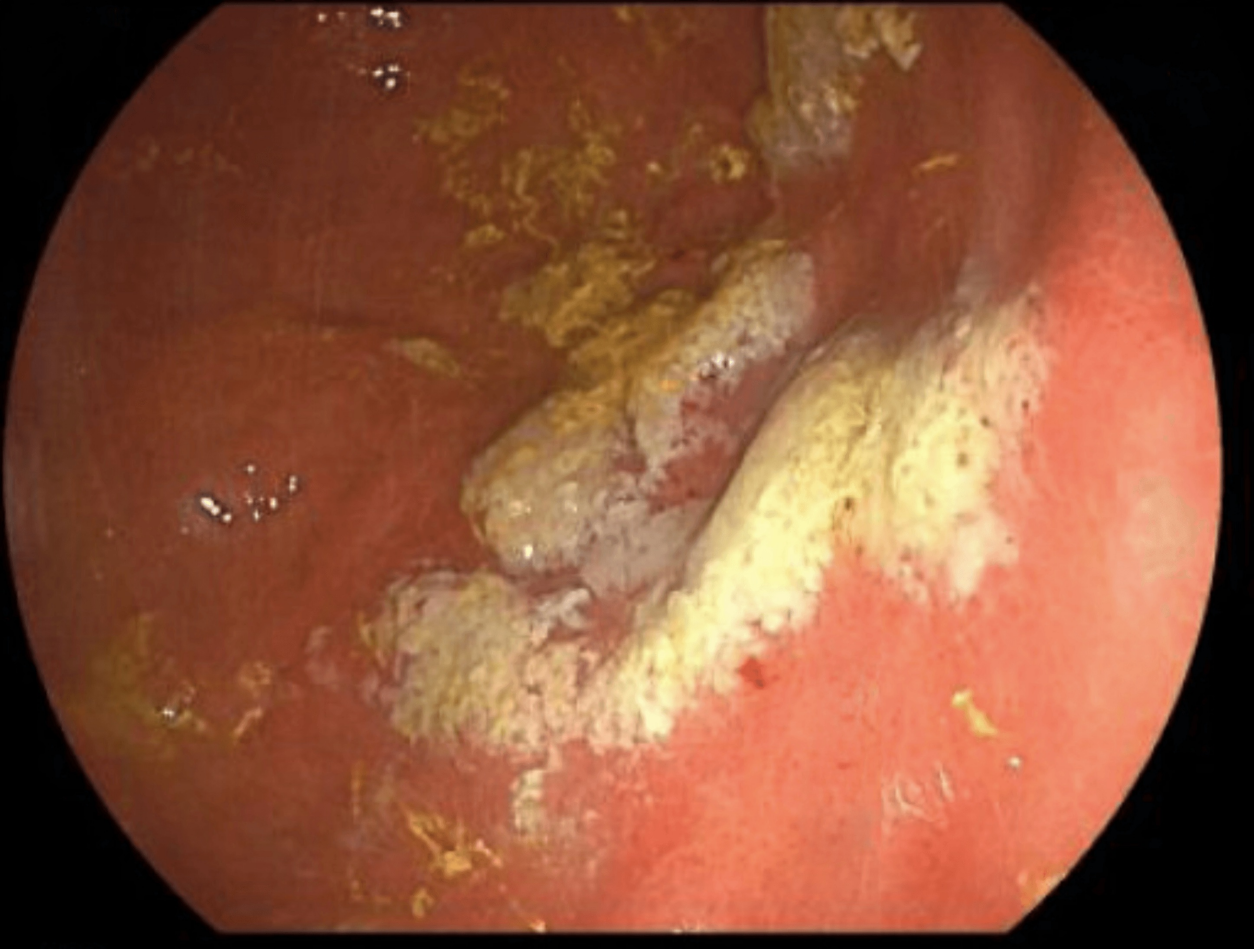
The index esophagogastroduodenoscopy (EGD) showed a 4x3 cm patch of exudate and scattered erosions in the gastric body.

On repeat EGD, the esophagitis was healed, but a new 4x3 cm patch of exudate and friable mucosa was seen within the gastric body (Figure 1).

**Figure 2:**
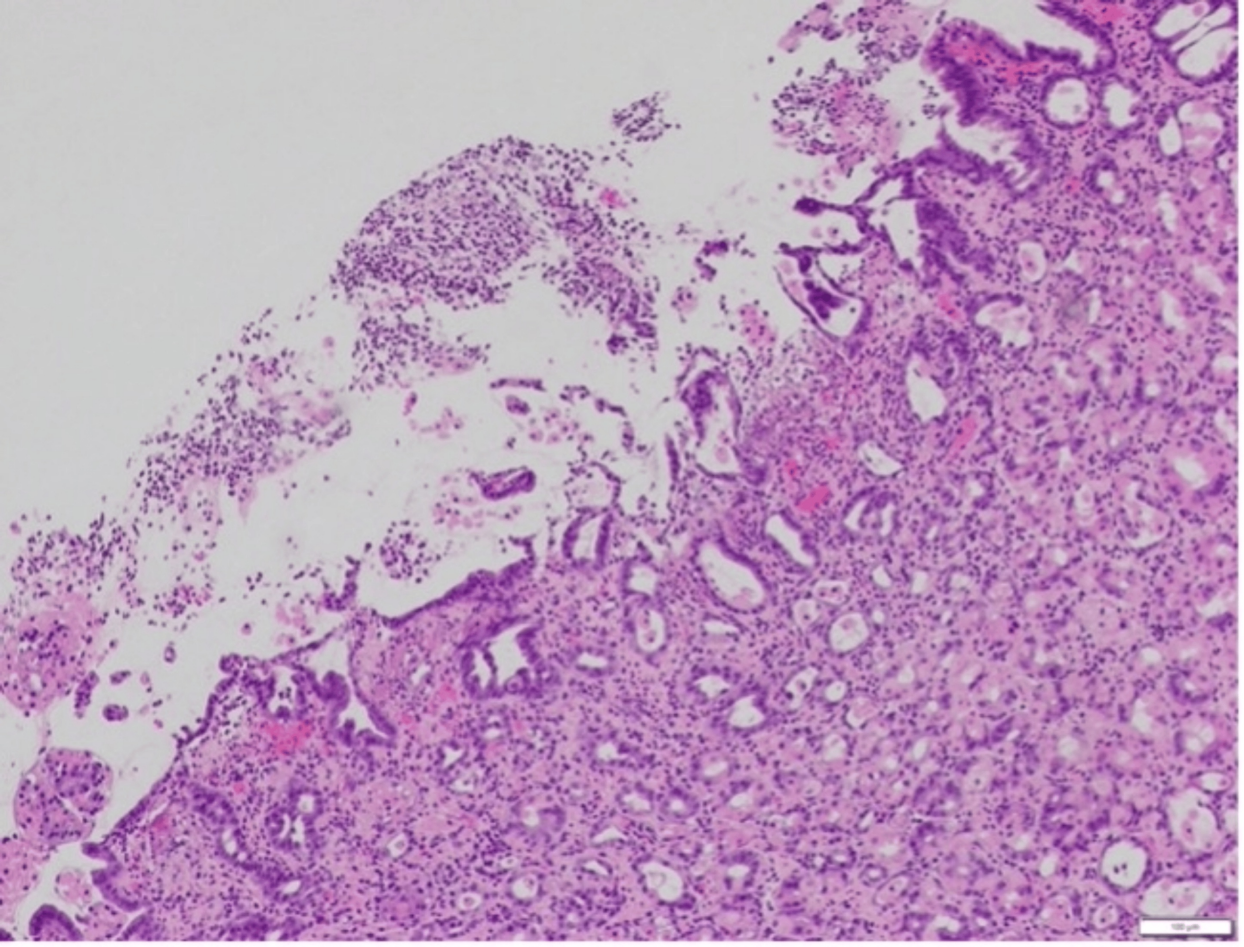
The H&E slide of gastric mucosa (10x magnification) Ulcerated gastric mucosa at the center with surrounding free-floating inflamed necrotic debris is noted. Gastric mucosa with normal architecture is seen at the peripheries.

Given the benign-appearing ulceration (Figure 2), this was thought to be medication-induced and related to hypercalcemia.

**Figure 3:**
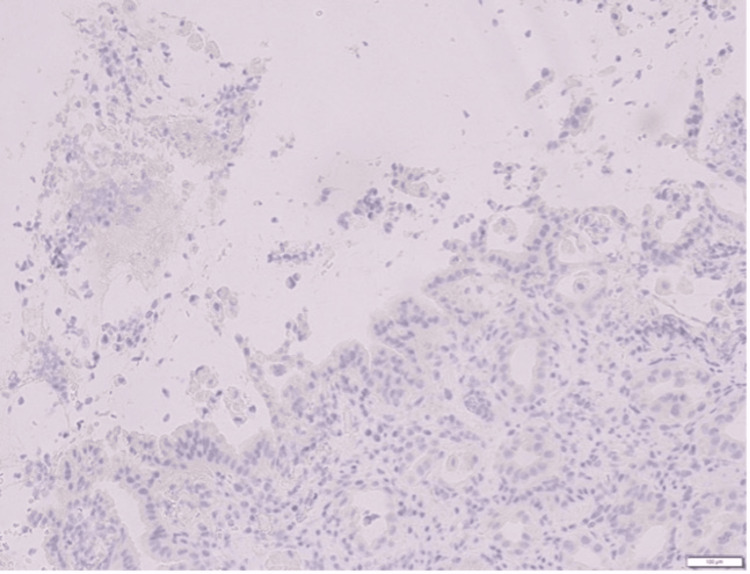
Immunohistochemistry (IHC) staining for Helicobacter pylori (20x magnification) was negative.

Immunohistochemistry (IHC) staining for *H. pylori* was negative (Figure 3), and the patient was not on long-term NSAID use.

**Figure 4:**
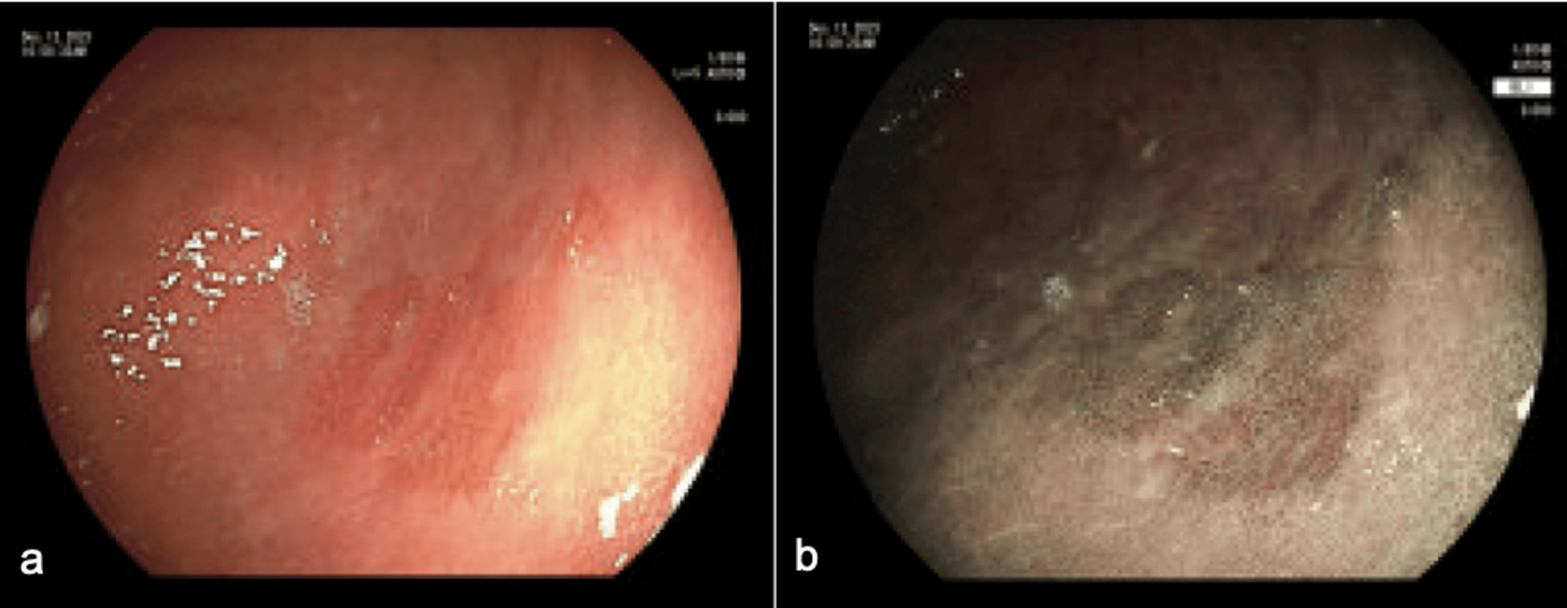
A third esophagogastroduodenoscopy (EGD) performed nine months after the index study under regular white light (a) and flexible spectral imaging color enhancement (FICE)(b). Overall, these demonstrated significant improvement in the area in question. Biopsy with chronic active inflammation, lymphoid aggregate, and reactive/regenerative changes; negative for *Helicobacter pylori*.

A thorough review of the patient's medication list found cinacalcet as the possible culprit. The patient was referred for parathyroidectomy to discontinue cinacalcet completely. However, given the complexity of the procedure, surgery was deferred, and the cinacalcet dose was decreased to 30 mg from 60 mg twice daily. The omeprazole was continued, and he was started on Carafate, with plans for surveillance EGDs. Serial EGDs at six and nine months showed improvement in non-bleeding gastric ulcers and erythematous mucosa in the gastric body (Figure 4).

**Figure 5:**
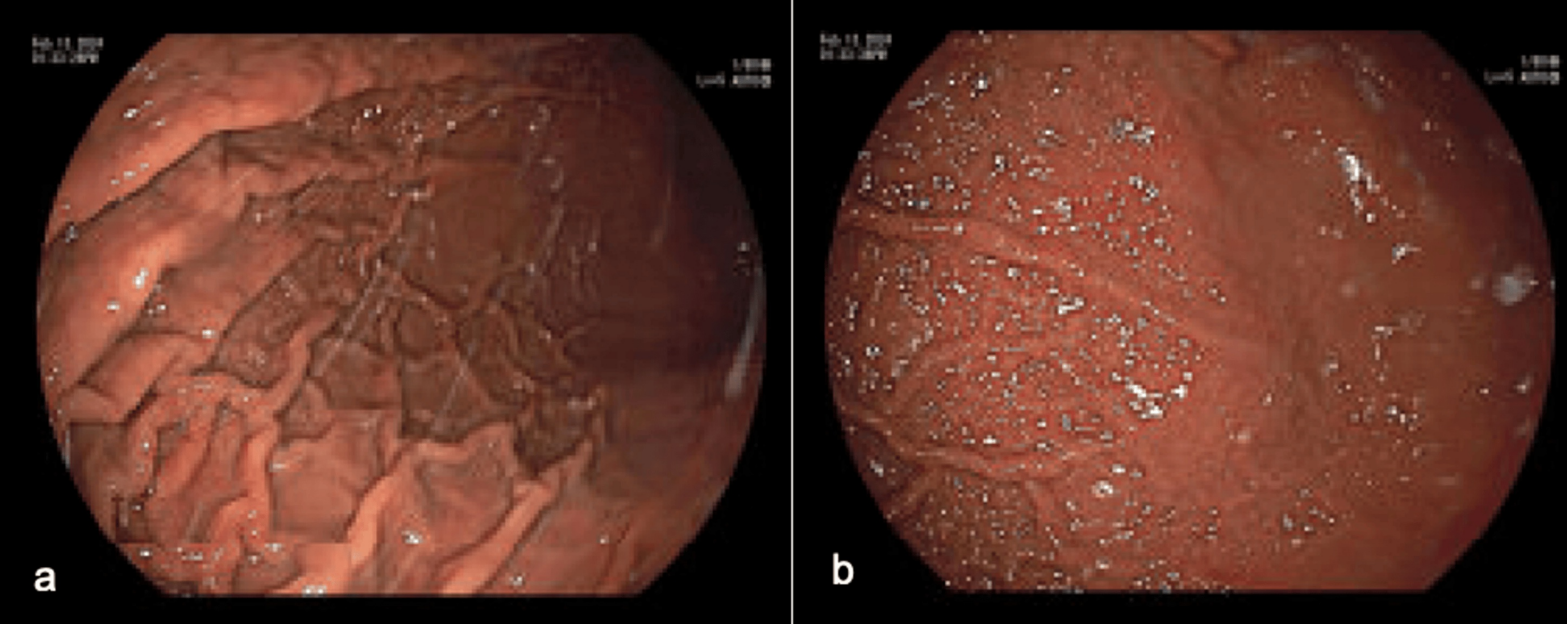
Fourth esophagogastroduodenoscopy (EGD) one year after the index study under regular white light. (a) and (b): Complete resolution of inflammation in the gastric body is noted.

After one year, there was a complete resolution of inflammation in the gastric body (Figure 5). 

## Discussion

There have been associations between primary hypercalcemia and peptic ulcer-related symptoms, with approximately 12% of patients with PHPT presenting with epigastric pain and nausea [14]. Cases of peptic ulcer perforation as the initial presentation of PHPT are extremely rare but have been reported in the literature. The current theory is that serum calcium activates gastrin cell calcium receptors, ultimately resulting in increased gastric acid production and PUD [15]. 

Our patient’s calcium and PTH levels were normal for many years leading up to his presentation. Therefore, it is likely a significant contribution from a different culprit was present in his gastric ulcer formation. The one identifiable source for this patient was cinacalcet, which is thought to activate the same gastrin cell calcium receptors that calcium does. In theory, gastrin cell calcium receptor activation by cinacalcet can then lead to the formation of PUD, especially over many years of activation. 

In a small, randomized placebo-controlled study, changes in gastrin levels in the cinacalcet-treated group were significantly higher than those in the placebo group [11]. The study suggested that activating CaSR in the stomach with an allosteric CaSR modulator, cinacalcet may lead to an increase in serum gastrin acid levels and basal gastric acid secretion. Interestingly, lower doses of cinacalcet of 15 to 30 mg daily for 11 days caused no GI symptoms in healthy participants. In contrast, dialysis patients with secondary hyperparathyroidism randomized to a 30 to 180 mg daily dose of cinacalcet had a 13% to 14% higher incidence of nausea and vomiting than those on placebo [16]. The most commonly attributed side effects of cinacalcet are nausea, vomiting, and generalized GI discomfort [11, 16-18]. While GI pathology arising as a result of long-term cinacalcet use is not well studied, this case suggests a dose-dependent association between cinacalcet use and GI side effects.

## Conclusions

This case report is one of the first to show that PUD may be induced by cinacalcet use. While no definite causation can be established, one year after the dose reduction of cinacalcet, complete resolution of gastric inflammation was seen on endoscopy. This case highlights that not only hypercalcemia from PHPT can cause PUD but also the treatment of hypercalcemia with cinacalcet. While cinacalcet is effective in treating hypercalcemia, more studies are needed to examine the effect of the long-term use of cinacalcet on the risk of gastric ulcer development.
